# Observation of Intramural Fibroid Expulsion on MRI after Uterine Artery Embolization

**DOI:** 10.1155/2021/7970894

**Published:** 2021-08-14

**Authors:** Hisakazu Matsushima, Ken Kageyama, Akira Yamamoto, Atsushi Jogo, Etsuji Sohgawa, Takehito Nota, Kazuki Murai, Satoyuki Ogawa, Mariko Nakano, Taro Shimono, Yukio Miki

**Affiliations:** Department of Diagnostic and Interventional Radiology, Graduate School of Medicine, Osaka City University, 1-4-3 Asahimachi, Abenoku, Osaka 545-8585, Japan

## Abstract

Uterine artery embolization (UAE) is a type of noninvasive treatment for symptomatic uterine fibroids. One of the complications of UAE is fibroid expulsion. Here, we report a case of a 45-year-old woman who underwent UAE for an intramural fibroid, which resulted in fibroid expulsion. To the best of our knowledge, there are only few reports of expulsion of intramural fibroids. The process of fibroid protrusion from the myometrium into the uterine cavity was depicted on magnetic resonance imaging (MRI) in this case. We discuss the risk factors and mechanisms of fibroid expulsion after UAE.

## 1. Introduction

Uterine fibroids are the most common type of female pelvic tumor. Ultrasound studies have shown that they are present in nearly 70% of adult women [1]. The procedure of uterine artery embolization (UAE) was introduced in 1995, and it has become one of the alternative treatments to surgery for symptomatic uterine fibroids in the past two decades. UAE is performed for both intramural and submucosal fibroids. The indications for UAE include the presence of symptoms, such as irregular bleeding, anemia due to excessive menstruation, abdominal bloating, incomplete voiding, contraindications to surgery, and ineffective drug treatment [2]. Contraindications to UAE include pregnancy, active pelvic infections, possible malignancy, pedunculated fibroid, cervical fibroid, and desire for future pregnancy [3, 4]. One of the complications of UAE is fibroid expulsion. Although expulsion of submucosal fibroids after UAE has been reported, expulsion of intramural fibroids is less common [5, 6]. We report a case of almost total expulsion of an intramural fibroid following UAE.

## 2. Case Presentation

**Figure 1:**
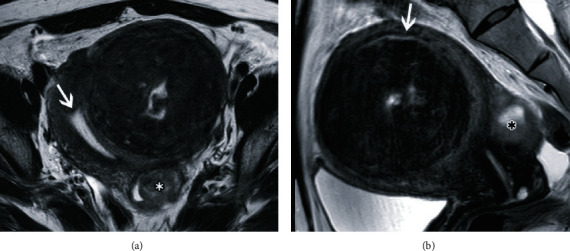
Magnetic resonance images before uterine artery embolization. (a) T2-weighted axial image demonstrates a large intramural fibroid. The central area has a high intensity, suggesting degeneration. The asterisk (∗) shows another degenerative uterine fibroid. (b) T2-weighted sagittal image demonstrates the endometrium (white arrow) compressed by the fibroid. The distance between the endometrium (white arrow) and the fibroid is very short. The asterisk (∗) shows another degenerative uterine fibroid.

**Figure 2:**
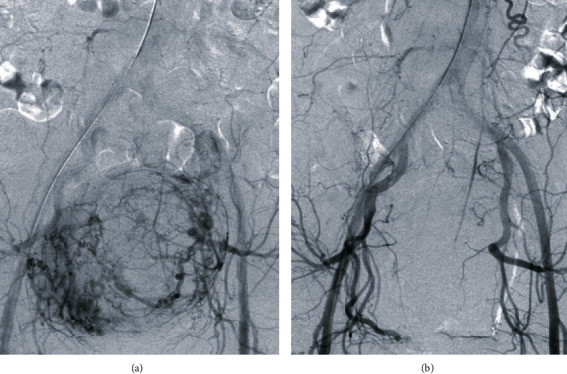
Digital subtraction angiography images. (a) Pre-embolization aortography demonstrates hypertrophic uterine arteries and staining of the fibroid. (b) Post-embolization aortography demonstrates stasis in the uterine arteries bilaterally.

**Figure 3:**
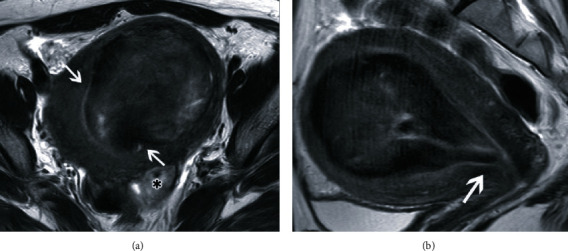
Magnetic resonance images 1 month after uterine artery embolization. (a) T2-weighted axial image demonstrates the fibroid protruding from the endometrium into the endometrial cavity (white arrows). The asterisk (∗) shows another degenerative uterine fibroid. (b) T2-weighted sagittal image demonstrates prolapse of the fibroid into the internal os (white arrow). A small portion of the fibroid is protruding into the uterine cavity.

**Figure 4:**
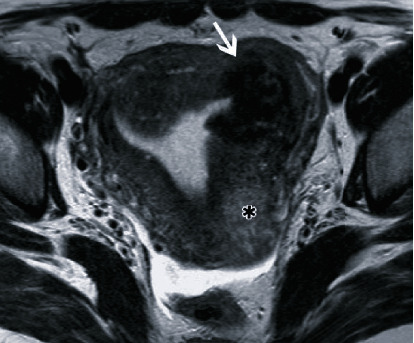
Magnetic resonance image 7 months following uterine artery embolization. T2-weighted axial image demonstrates a residual fibroid (white arrow) in the myometrium. The irregularity of the endometrium adjacent to the fibroid is shown. The asterisk (∗) shows another degenerative uterine fibroid.

**Figure 5:**
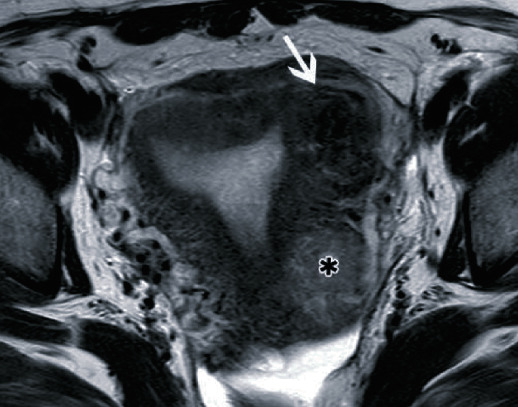
Magnetic resonance image 11 months following uterine artery embolization. T2-weighted axial image demonstrates further shrinkage of the residual fibroid (white arrow) and improvement of the endometrial irregularity. The asterisk (∗) shows another degenerative uterine fibroid.

A 45-year-old woman presented with excessive menstruation for 3 years and was diagnosed with a uterine fibroid. Six courses of gonadotropin-releasing hormone (GnRH) were administered, but there was no improvement in her condition. Hence, the patient visited our hospital to undergo UAE. MRI demonstrated an 86 × 85 × 77 mm intramural fibroid in the uterine myometrium on the left side. On T2-weighted image (T2WI), the fibroid had a homogeneously low signal and was present in close proximity to the endometrium (Figure 1). Vascular access was achieved via the right common femoral artery using a 4Fr sheath (Medikit Co. Ltd., Tokyo, Japan) under local anesthesia. At first, we performed aortography using a 4Fr Universal Flush catheter (Cordis Japan Co. Ltd., Tokyo, Japan) to confirm staining of the uterine fibroid (Figure 2(a)). Next, a 4Fr Mohri's catheter (Medikit, Tokyo, Japan) was inserted into the internal iliac arteries bilaterally. Then, selective catheterization of the uterine arteries bilaterally was achieved with a 2.8Fr microcatheter (Carnelian, Tokai Medical Products, Aichi, Japan). Embolization of both uterine arteries was performed with tris-acryl gelatin microspheres (Embosphere; Nippon Kayaku Co. Ltd., Tokyo, Japan), 500-700 *μ*m in size on the right side and 500-700 and 700-900 *μ*m in size on the left side. After embolization, we confirmed disappearance of staining of the fibroid (Figure 2(b)). The procedure was completed without any intraoperative complications, and the patient had an uneventful postprocedure course, as assessed at the 1-week follow-up. At 1 month after UAE, routine MRI showed that the fibroid shrunk to 95 × 75 × 70 mm; however, it was protruding into the cervical canal beyond the endometrium (Figure 3). Four days later, fibroid expulsion occurred, followed by a foul-smelling secretion and bleeding. These symptoms spontaneously disappeared without any therapy within one week. At 7 months after UAE, MRI showed a small residual fibroid remaining in the uterus after fibroid expulsion and the irregularity of the endometrium (Figure 4). Mild menorrhagia was still present; however, it was well controlled with hormone treatment. There were no signs of infection throughout the course of fibroid expulsion or thereafter. At 11 months after UAE, MRI showed further shrinkage of the residual fibroid and improvement of the endometrial irregularity (Figure 5).

## 3. Discussion

Fibroid expulsion typically occurs with submucosal fibroids, with expulsion of intramural fibroids being an uncommon event [5, 6]. In this case, the bulk of the intramural fibroid protruded into the uterine cavity after UAE, ultimately leading to fibroid expulsion. The process of fibroid protrusion from the myometrium into the uterine cavity was visualized on MRI in this case.

Fibroid expulsion is an important complication after UAE. The other major complications of UAE include pulmonary embolism, necrosis of the uterus, sepsis associated with endometritis, buttock/leg ischemia, and premature ovarian failure [2, 3]. Fibroid expulsion often occurs within 3 months after UAE, although it might occur as much as 4 years later. The frequency of fibroid expulsion is estimated to be 3-5%, with most of the cases involving submucosal fibroids. There are two types of fibroid expulsion: bulk type, in which the entire fibroid protrudes into the uterine cavity, and sloughing type, in which the fibroid liquifies and merges with the endometrium and is chronically excreted in small amounts. Bulk-type expulsion of intramural fibroids after UAE is extremely rare [7]. In this case, the majority of the fibroid protruded into the uterine cavity, suggesting that it underwent bulk-type expulsion.

The exact mechanism of intramural fibroid expulsion is not clear. The fibroids are generally absorbed by the myometrium after UAE [2]. The suggested hypothesis of fibroid expulsion is that contraction of the uterus results in protrusion of the intramural fibroid toward the submucosa [8]. Two major mechanisms have been reported to be involved in uterine contraction and fibroid expulsion. Marret et al. considered that the inflammatory reaction secondary to fibroid necrosis causes contractions [5]. Mailli et al. considered that ischemic changes due to embolization damage the endometrium, leading to expulsion [9]. Recent study revealed that uterine fibroids could migrate from the myometrium to the submucosa after UAE and analyzed the predictive indicators of migration. Of 35 intramuscular uterine fibroids, 13 cases showed migration of the fibroids after UAE. Among these, eight migrated to the submucosa, two were partially expelled into the uterine cavity, and three were completely expelled into the uterine cavity. The authors identified two risk factors for fibroid migration after UAE: large fibroid (mean maximum diameter = 103 mm, range = 51‐181 mm) and short distance between the endometrium and the edge of the fibroid (mean minimal distance = 1.7 mm, range = 1.0‐2.4 mm) [9]. In our case, the maximum diameter and the minimal distance were 86 mm and 1 mm, respectively; thus, we speculated that the fibroid was prone to migration.

Fibroid expulsion can lead to further complications, such as necrotic tissue infection. Interruption of local blood flow following UAE might result in sepsis. When conservative antibiotics therapy does not work well for infection after UAE, hysterectomy is required [2, 5]. Fortunately, in this case, there were no signs of infection. The patient showed temporary secretion and bleeding at the time of fibroid expulsion, followed by spontaneous improvement of these symptoms with conservative follow-up.

At 7 months after UAE, MRI showed the irregularity of the endometrium adjacent to the fibroid. Previous reports also showed irregularities of the endometrium on MRI after fibroid expulsion [4, 9]. It is speculated that the irregularity was associated with fibroid expulsion; however, the exact mechanism is unknown. In our case, at 11-month follow-up, MRI showed reduction of the residual fibroid and improvement of the endometrial irregularity. Further reduction of the residual fibroid might lead to improvement of the endometrial irregularity.

In this case, GnRH was administered prior to UAE. There have been few reports of submucosal fibroids treated with GnRH undergoing fibroid expulsion [10, 11]. However, we could not find any reports of expulsion of an intramural fibroid after treatment with only GnRH. Therefore, although we believe that UAE was the major factor in fibroid expulsion in our patient, it is also possible that preoperative GnRH administration caused certain changes in the endometrium that increased the likelihood of fibroid expulsion. Hence, we cannot definitively state that UAE alone was involved in fibroid expulsion in our patient.

In conclusion, intramural fibroids can undergo expulsion after UAE. Fibroid expulsion should be noted as a potential complication after UAE, even if the fibroid is preoperatively located in the myometrium.

## Data Availability

The data that support the findings of this study are available from the corresponding author.
